# Survival of critically ill patients with haematological malignancies compared with patients without haematological malignancy

**DOI:** 10.1186/cc12453

**Published:** 2013-03-19

**Authors:** R Pugh, P Hampshire, P Hajimichael

**Affiliations:** 1 Glan Clwyd Hospital, Rhyl, UK; 2 Royal Liverpool University Hospital, Liverpool, UK; 3 Christie Hospital, Manchester, UK

## Introduction

Critically ill patients with haematological malignancies (HM) have high hospital mortality [1]. Severity of illness scores may underestimate mortality in such patients [2].

## Methods

Data collection was conducted at three hospitals from 2008 to 2011. Patients with any active HM condition were matched with two control patients at two hospitals and with one control at Christie Hospital. Control patients had the same APACHE II (within 2 points) and admission diagnosis, but no HM. Readmissions and planned surgical cases were excluded.

## Results

A total of 163 patients with HM were compared with 237 control patients. Seventy-four admissions with HM were identified at two hospitals, and each was matched with two control patients. Eighty-nine admissions with HM from Christie Hospital were identified. These were matched with 89 controls. Patients with HM spent significantly longer in hospital before ICU admission (Table 1). Unit and hospital mortality rates were not statistically different between patients with HM and without HM (Table 2).

**Table 1:** Patient characteristics

	Haematology	Controls	*P *value
Age, mean (SD)	56.5 (15.1)	57.1 (15.7)	0.717
APACHE II, mean (SD)	22.7 (6.1)	23.1 (6.0)	0.484
ICU LOS (median)	3.2 (1.8 to 6.2)	3.1 (1.3 to 8.4)	0.948
LOS prior (median)	6.5 (2 to 17)	2.0 (0 to 7)	<0.0001

**Table 2:** Unit and hospital mortality

	Patients	**ICU mortality**,	**Hospital mortality**,
	(*n*)	*n *(%)	*n *(%)
Haematology	163	52 (31.9%)	81 (49.7%)
Controls	237	73 (30.8%)	99 (41.8%)
*P *value		0.827	0.126

## Conclusion

Unit mortality of critically ill patients with HM was similar to those without HM. Hospital mortality in patients with HM was higher than those without HM, although not statistically significant. Severity of illness at presentation to critical care is the main determinant of outcome in patients with HM.
